# Simultaneous Regularity Contrast and Luminance Polarity

**DOI:** 10.3390/vision9010023

**Published:** 2025-03-13

**Authors:** Frederick A. A. Kingdom, Hua-Chun Sun, Elena Gheorghiu, Martin S. Silva

**Affiliations:** 1 McGill Vision Research, Department of Ophthalmology and Visual Sciences, Montreal General Hospital, Montreal, QC H3G 1A4, Canada; martin.sellier@mail.mcgill.ca; 2 Department of Psychology, Justus Liebig University, 35394 Giessen, Germany; hua-chun.sun@psychol.uni-giessen.de; 3 Department of Psychology, University of Stirling, Stirling FK9 4LA, UK; elena.gheorghiu@stir.ac.uk

**Keywords:** texture, regularity, surround contrast, assimilation, luminance polarity, spatial frequency, kurtosis

## Abstract

Texture regularity, for example, the repeating pattern of a carpet, brickwork, or tree bark, is a ubiquitous feature of the visual world. The perception of regularity has generally been studied using multi-element textures whose regularity is manipulated by the addition of random jitter to the elements’ nominal positions. Here, we investigate the selectivity of regularity perception for the luminance contrast polarities of the elements. Our psychophysical tool was simultaneous regularity contrast, or SRC, the phenomenon in which the perceived regularity of a central test texture is shifted away from that of the surrounding regularity. Stimuli were composed of arrays of dark and/or white Gaussian elements. Surround and center test textures consisted of either the same (“congruent”) or opposite (“incongruent”) polarities. In addition, we tested a “mixed” condition consisting of a random mixture of polarities in both the surround and test. The perceived regularity of the test was measured using a match stimulus with the same polarity dimension as the test. The regularity of the match stimulus was adjusted on each trial using a forced-choice staircase procedure and the point-of-subjective equality between the match and test regularities was estimated from the resulting psychometric functions. SRC was observed in both congruent and incongruent conditions, but with the mixed condition, the perceived regularity of the test was shifted toward rather than away from the surround regularity, an example of assimilation, not contrast. The analysis revealed no significant difference in the magnitude of SRC between the congruent and incongruent conditions, suggesting that SRC could be mediated solely by polarity agnostic mechanisms, although there are other possible explanations for the “null” result. However, trend analysis using a non-linear (sigmoidal-shaped) function indicated a significant difference between the congruent and incongruent conditions, which, together with the mixed polarity results, suggests the presence of at least some polarity selective mechanisms. Previous reports have suggested that regularity perception is encoded by the “peakedness” in the distribution of spatial-frequency-tuned linear filter responses. We modelled SRC quantitatively by incorporating peakedness with spatial-frequency-selective surround inhibition and found that the model gave a good account of the SRC data. Possible reasons for the assimilation effect—with the mixed polarity condition are discussed.

## 1. Introduction

Naturally occurring, as well as laboratory textures, can be defined along different dimensions, and how texture dimensions are represented in the brain continues to engage the research community. One such dimension is texture regularity, defined as the degree of orderliness or repetitiveness in the positions of texture elements. Texture regularity is also a type of symmetry termed “translational” [1,2,3,4,5,6]. Patterns with a high degree of texture regularity are ubiquitous in the visual world, for example, in the repetitive patterns of carpets, brickwork, and tree bark and in experimental stimuli comprised of micropatterns arranged quasi-periodically. Regular structures carry important information about the etiology and biological function of natural and artificial surfaces [1,2,7]. In keeping with the idea that “if it is adaptable it must exist”, texture regularity has been shown to be adaptable [8,9] and subject to the contrasting influence of a surround with different regularity, termed simultaneous regularity contrast, or SRC [10]. Regularity has been found to influence several types of visual processing, such as texture segregation [11], contour detection [12], numerosity perception [13,14,15], and surface slant perception [2]. Perceived regularity can also be influenced by adaptation to other dimensions, such as texture density [16]. However, much remains to be discovered about how the visual system encodes regularity information and how perceived regularity is influenced by context.

In this communication, we consider the extent to which the perception of texture regularity (from now on just regularity) in dot textures is selective for luminance contrast polarity (from now on just polarity) of the elements, i.e., whether the elements are white or black. Selectivity for polarity has been observed in a number of visual dimensions, most notably texture segregation [17,18,19,20,21,22,23] but also simultaneous contrast/contrast [24,25], density perception [26,27], and shape perception [28,29], with the tilt after-effect seemingly the lone exception [30,31]. Of particular relevance here is the study by Yamada et al. [9]. They measured adaptation-induced after-effects in perceived randomness as a function of whether the polarities of the adaptor and test were congruent (same polarity) or incongruent (opposite polarity) and found no significant difference, concluding that randomness perception was not selective for polarity. Assuming that randomness is the inverse of regularity, the same conclusion presumably holds for regularity.

It is widely believed that luminance decrements are detected by neurons with “OFF-center” and luminance increments by “ON-center” receptive fields [32]. If texture regularity perception is selective for polarity, this implies that the ON- and OFF-center responses are fed into separate regularity-sensitive streams with each independently contributing at the decision stage. On the other hand, if regularity perception is agnostic to polarity, this would imply that the ON and OFF responses are combined prior to the decision stage.

Our tool for measuring regularity perception is simultaneous regularity contrast, or SRC. SRC is the phenomenon in which the regularity of a surrounding texture influences the perceived regularity of a central test texture with different regularity [10]. Given that studies have tended to show that adaptation and surround induction behave similarly, we would expect, on the basis of Yamada et al. [9], that SRC is polarity agnostic. On the other hand, the sheer number of studies described above showing polarity selectivity would suggest that SRC is polarity selective. The first aim of the present study is to test between the two hypotheses.

**Figure 1:**
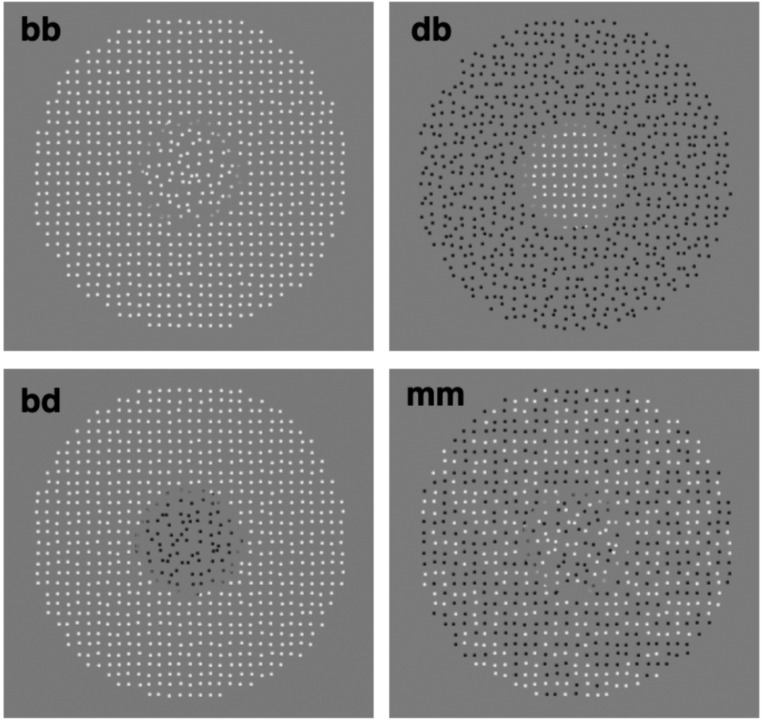
Example stimuli used in the experiments. **b** = bright Gaussian; **d** = dark Gaussian; **m** = mixture of bright and dark Gaussian. First letter refers to the surround inducer, second letter the central test patch. **bb** is referred to as a congruent condition, while **db** and **bd** are referred to as incongruent conditions and **mm** the mixed condition.

Example stimuli are shown in Figure 1. If regularity-sensitive mechanisms are selective for polarity, the magnitude of SRC would be expected to be greater when the test and surround textures consist of elements with congruent compared to incongruent polarities. We might also expect that the magnitude of SRC will be relatively small for the “mixed” (mm) random-polarity textures in Figure 1 on the grounds that regularity-sensitive mechanisms will be poorly stimulated.

### Models of Regularity Perception and Simultaneous Regularity Contrast

The observation that different degrees of regularity can be effortlessly discriminated (see Figure 1) has led investigators to suggest that pattern regularity is, like other texture dimensions mediated by Fourier (or wavelet) energy-based operations [8,10,33,34]. Ouhnana et al. [8] were the first to suggest that pattern regularity was encoded by the “peakedness” in the population response of channels selective for spatial frequency (SF). This idea has been endorsed by subsequent studies of the regularity after-effect [9], simultaneous regularity contrast [10], regularity discrimination [33,35], and regularity scaling [33,34,36], with the last of these studies suggesting that peakedness in the response distribution of orientation channels is also likely to be involved.

Sun et al. [10] opined that SRC results from within-SF-channel surround-to-center inhibition. They suggested that the inhibition primarily occurs for any SF channel whose amplitude is higher in the surround compared to the center; that is, the inhibition is “unidirectional”, as found in most previous studies of simultaneous contrast/contrast (reviewed by [37], but see to the contrary [38]. Sun et al. [10] argued that, in principle, unidirectional surround inhibition could translate to bidirectional SRC. However, Sun et al. [10] did not attempt to quantitatively model their idea, so it is necessarily speculative. The second aim of the present study is, therefore, to formulate a quantitative model of SRC based on the ideas put forward by Sun et al. [34].

## 2. Methods

### 2.1. Observers

Seven observers took part in the study: six undergraduate volunteers and one investigator. All experiments were conducted in accordance with the Declaration of Helsinki and the Research Institute of the McGill University Health Center (RI-MUHC) Ethics Board. Observer initials on graphs have been anonymized in accordance with requirements of the (RI-MUHC) Ethics Board.

Example stimuli are shown in Figure 1. Each is an array of bright “b” and/or dark “d” Gaussian elements with a standard deviation of 0.05 deg and Weber contrast of 0.85, presented on a mid-grey background of 61 cd/m^2^. The Gaussian elements were clipped at a diameter of 0.25 deg and placed on a 35 × 35 notional grid with an average center-to-center separation of 0.5 deg. Each element was independently jittered in both x and y directions by an amount drawn randomly from a uniform distribution centered on its notional position with a range that determined the degree of irregularity of the texture (see below).

### 2.2. Stimuli

All experiments were conducted using a Dell Precision T1650 PC with a VISaGe graphics card (Cambridge Research Systems (CRS), Rochester, Kent, UK). The visual stimuli were displayed on a gamma-corrected Sony Trinitron Multiscan F500 flat-screen CRT Monitor. Stimulus generation and experimental control employed custom software written in C/C++ (Embarcadero Builder v.11.0) with embedded VISaGe subroutines (v. 8.3). During the experiments, observers were seated in a light room and their responses were recorded via a keypad.

For the surround stimulus, the Gaussian elements were located in a circular region with an outer diameter of 15 deg and an inner diameter of 5 deg. The test stimulus was contained within the surround and had a diameter of 5 deg. To help observers perceptually segregate test and surround, the outermost elements of the test were blurred by multiplication with a half-Gaussian with a standard deviation of 0.05 deg and amplitude of unity. The origin and, hence, maximum value of the blurring Gaussian lay 2.5 deg from the center of the stimulus, tapering downwards towards its outer edge at which point it was clipped (see Figure 1). The match stimulus was the same as the test but with no surround and a regularity that was adjustable.

Positional jitter (from now on just jitter) was applied to the elements in the test, surround, and match stimulus. The test jitter was fixed throughout the experiment at 0.2 deg. There were nine surround jitter levels: 0.0, 0.05, 0.1, 0.15, 0.2, 0.25, 0.3, 0.4, and 0.5 deg. Thus, four surrounds had less than and four surrounds greater than the jitter of the test. The jitter of the match stimulus was adjusted during a session to match the perceived regularity of the test (see below). There were five surround/test combinations: dd, db, bd, bb, and mm, where the first letter of each pair denotes the surround polarity and the second letter the test polarity. The bb and dd conditions are termed congruent conditions, db and bd incongruent conditions, and mm the mixed polarity condition.

### 2.3. Procedure

**Figure 2:**
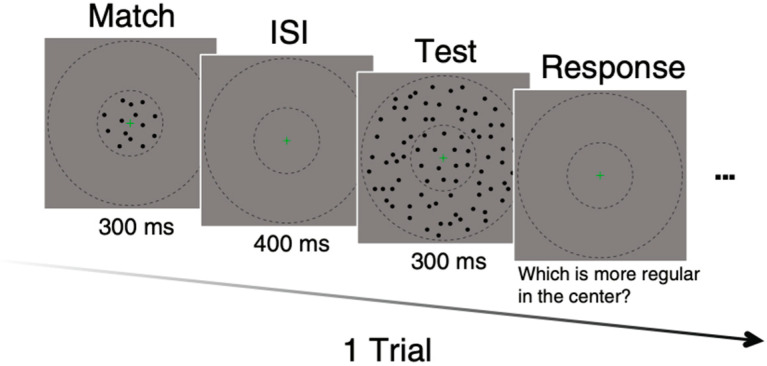
Stimulus sequence and task for a single trial for condition dd. The dashed circles represent the center and surround regions but were not present during the experiment.

The stimulus sequence on each trial is shown in Figure 2. Stimuli were presented for 300 msec in a raised cosine envelope with an interstimulus interval of 400 ms. Two interleaved one-up-one-down staircases were employed to establish the PSE (point of subjective equality) between the test and match regularities. The task on each trial was to decide which of the two center stimuli had the higher perceived regularity. As this was an appearance-based task, no feedback was provided.

A green fixation cross 8.8 arcmin in diameter was presented in the center of the stimulus throughout to aid fixation. We also applied 9.7 arcmin of jitter independently to the x and y locations of the stimulus as a whole to minimize potential afterimages, which were most likely to occur with the highly regular textures. Observers were instructed to fixate throughout the experiment and respond only to the perceived regularity of the center area, regardless of the surround.

At the start of the session, the jitter of the match stimulus was set to a random value for each staircase in the range 0.16–0.24 deg, i.e., centered on the test jitter of 0.2 deg. The match stimulus jitter was adjusted on every trial of each staircase depending on the previous response by ±0.04 deg for the first 3 trials and ±0.02 deg thereafter. If the response was “first stimulus more regular”, jitter was added to the match stimulus, whereas if the response was “second stimulus more regular”, jitter was subtracted from the match stimulus for the next staircase trial.

Each observer performed all nine surround jitter levels, with the test fixed at 0.2 deg jitter for all conditions, making a total of 5 surround/test polarity combinations × 9 surround jitters = 45 conditions. Only one condition was performed during each session of 50 trials (25 trials per staircase), and observers performed 3 sessions per condition, making 150 trials per condition. Prior to the start of the main experiment, observers performed 100 practice trials to familiarize themselves with the task. Observer sessions lasted a maximum of 60 min to avoid fatigue.

### 2.4. Analysis

The trials were combined across sessions for each condition, and the proportion of trials in which the test was chosen as more regular was plotted against the jitter level of the match stimulus. Using a maximum-likelihood criterion implemented by routines from the Palamedes toolbox [39], the psychometric functions (PFs) were fitted with a logistic function with the PSE estimated at the 50% level along with the slope of the PF. Errors on both estimates were calculated by bootstrap analysis (1000 samples).

To compare across surround regularity conditions, the data were normalized by subtracting from each PSE the match for the 0.2 deg surround jitter condition, i.e., the condition in which surround and test jitter were the same. The effect of this normalization is to re-center the PSEs around zero, such that positive values indicate that the surround has increased the perceived irregularity (or decreased perceived regularity), while negative values indicate the opposite. The normalization was applied separately to each center/surround polarity combination and for each observer. Differences in normalized SRC were assessed with repeated-measures ANOVAs (SPSS v.29) and trend analysis (GraphPad Prism v.10). For the ANOVAs, the Greenhouse–Geisser correction was applied, whilst post-hoc tests (paired-samples *t*-tests) were performed with a Bonferroni correction for multiple comparisons.

## 3. Results

### 3.1. Points of Subjective Equality (PSEs)

**Figure 3:**
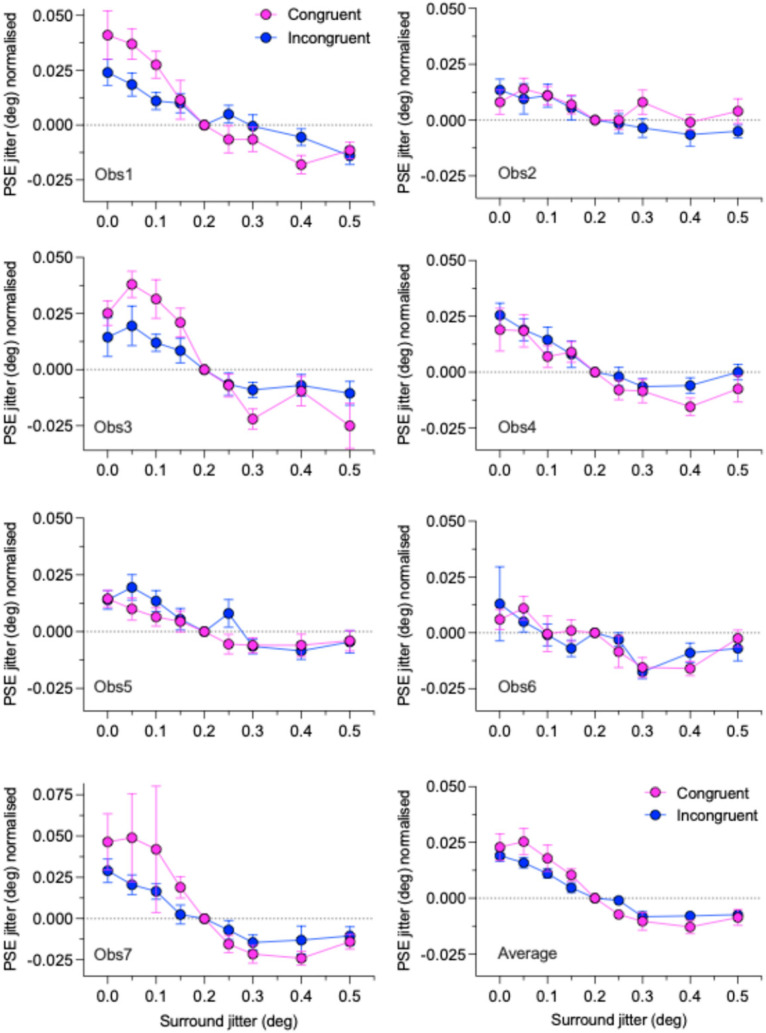
Normalized PSEs for the congruent and incongruent conditions for 7 observers (Obs) and their average (bottom right plot) are plotted against the jitter of the surround. PSEs for the two congruent (dd and bb) and two incongruent (bd and db) conditions have been averaged and then normalized to the PSE for the 0.2 deg surround jitter condition. Error bars on each observer’s data are the root mean square bootstrap errors per pair of conditions. Error bars for the observer average (bottom right panel) are standard errors calculated across observers.

Figure 3 shows normalized PSE jitters for the congruent (average of dd and bb) and incongruent (average of db and bd) polarity conditions for each observer, along with the across-observer averages. There are notable differences between observers. Whereas all observers show a positive-to-negative trend, indicative of SRC, only 3 observers (1, 3, and 7) appear to show a difference between the congruent compared to incongruent conditions. The across-observer averages confirm the presence of SRC in all conditions, with a modest average difference between the congruent and incongruent conditions.

**Figure 4:**
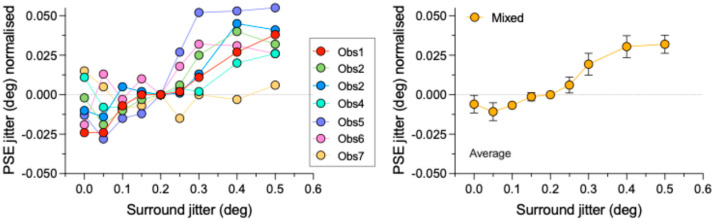
Normalized PSEs for the mixed polarity “mm” condition. Left panel shows individual observer data, right panel the average across observers. Error bars are not shown for the individual observer data to avoid cluttering. Error bars in the right panel are standard errors calculated across observers.

Figure 4 plots the results for the mixed polarity (“mm” in Figure 1) condition, with the left panel showing PSEs for all 7 observers and the right panel their average. The mixed polarity results are quite different from the unmixed polarity results shown in Figure 3 in that they appear to show a reversal of SRC, i.e., an “assimilation” of the test regularity; that is, a shift in the test toward rather than away from the regularity of the surround.

### 3.2. Evidence for Simultaneous Regularity Contrast (SRC)

**Figure 5:**
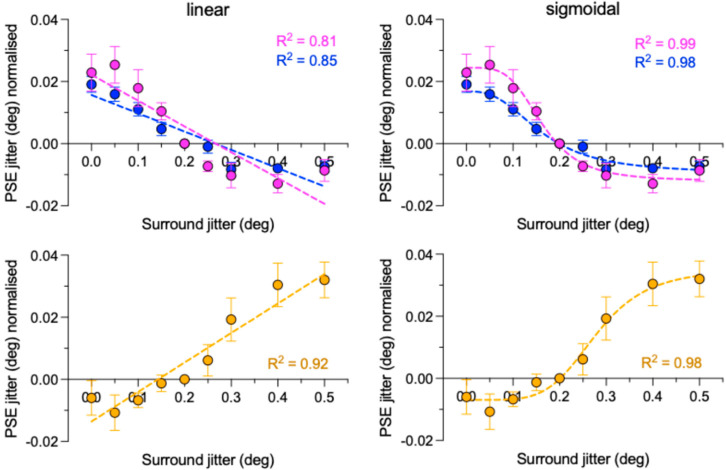
Trend analyses. Continuous lines are best fits to the congruent (magenta), incongruent (blue) and mixed (orange) conditions, using the across-observer averages. Left panels are for linear regression line fits, right panels for non-linear sigmoidal regression fits. Coefficients of determination R^2^ are given for each fit. Error bars are standard errors calculated across observers. See text for further details.

The positive-to-negative downward trend of PSEs as a function of surround jitter, i.e., irregularity, for both congruent and incongruent conditions, is evidence for “bidirectional” SRC. In other words a relatively high surround irregularity causes a lower test irregularity to appear even lower and a relatively low surround irregularity causes a higher test irregularity to appear even higher. To confirm the presence of SRC, we carried out two analyses: a trend analysis and a series of one-sample *t*-tests, both on the normalized data. For the trend analysis, we fitted a linear regression line to the congruent and incongruent data, as shown in the top left panel of Figure 5, and tested whether the slopes of both fitted functions were significantly different from zero. The analysis revealed that they were for both congruent (F(1,7) = 29.5, *p* = 0.001) and incongruent F(1,7) = 40.8, *p* = 0.0004) conditions.

In addition, we carried out directional one-sample *t*-tests on the normalized SRC data to examine whether SRCs were significantly greater/smaller than zero for each surround jitter condition and irrespective of the center-surround polarity combination. PSEs for surround jitters less than 0.2 were all significantly greater than 0 (*p*’s < 0.001), and PSEs for surround jitters greater than 0.2 were all significantly less than 0 (*p*’s < 0.021). Full details of the *t*-tests, including effect sizes and confidence intervals, are given in Appendix A.

### 3.3. Differences Between Polarity-Congruent and Polarity-Incongruent SRCs

Turning now to the main question of the study, namely whether the congruent and incongruent conditions produced different magnitudes or patterns of SRC, we first conducted a three-way repeated measures ANOVA on the normalized PSE data, with the factors Polarity Combination (congruent vs. incongruent), Surround Sign (less vs. greater than 0.2), and Surround Jitter (4 levels). The analysis revealed significant main effects of Surround Sign (F (1,6) = 27.7, *p* = 0.002, $\eta_{p}^{2}$ = 0.82) and Surround Jitter (F(2.16,12.98) = 12.56, *p* < 0.001, $\eta_{p}^{2}$ = 0.68), and a significant interaction effect between Surround Sign and Surround Jitter F(2.04, 12.27) = 8.31, *p* = 0.005, $\eta_{p}^{2}$ = 0.58). The main effect of Polarity Combination (F(1, 6) = 0.75, *p* = 0.42, $\eta_{p}^{2}$ = 0.11) and all other two-way and three-way interaction effects were however not significant (all *p*’s > 0.105), suggesting that SRC is agnostic to the particular combination of center and surround polarity. Bonferroni-corrected post-hoc comparisons (paired-sample *t*-tests) between congruent and incongruent conditions revealed that only the 0.25 surround jitter condition was significant (*p* = 0.023, 95% CI [−0.011 −0.001])). All other pairwise comparisons were non-significant (all *p*’s > 0.065).

The linear fits to the data shown in Figure 5 show slightly steeper negative slopes for the congruent compared to incongruent conditions, consistent with a greater magnitude of SRC for the former condition. However, as with the ANOVA results, the analysis reveals that the differences between the slopes of the two conditions (F(1,14) = 1.845, *p* = 0.1959) and between their intercepts (F(1,15) = 0.1939, *p* = 0.6659) were not significant.

We also fitted sigmoidal functions to the data, shown in the top right-hand panel of Figure 5. The sigmoidal fits are better than the linear fits, as shown by their relative R^2^ values. We tested whether a single or separate sigmoidal curve fitted the congruent and incongruent data and found that separate fits fared better, revealing that the congruent and incongruent data are significantly different (F (4, 10) = 5.85, *p* = 0.0109), in keeping with the idea that SRC is *not* agnostic to the particular combination of center and surround polarity. We discuss these results in the Section 4.

### 3.4. Evidence for Assimilation with the Mixed Polarity Stimuli

The slope of the linear regression line fit for the mixed condition in the lower left panel of Figure 5, which is significantly greater than zero, demonstrates assimilation: F(1,7) = 76.98, *p* < 0.0001. One-sample *t*-tests revealed that the amount of assimilation was significantly less than zero for surround jitters of 0.1 and marginally significant for 0.05, while significantly greater than zero for 0.3, 0.4, and 0.5 surround jitters. All other one-sample *t*-tests were found not significant (all *p*’s > 0.110). Details of the *t*-tests for the mixed condition are provided in Appendix B.

### 3.5. Psychometric Function Slopes

**Figure 6:**
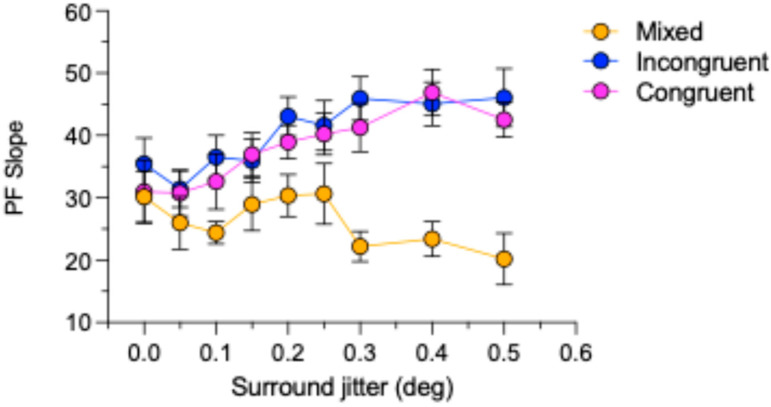
Psychometric function (PF) slopes for the match PSEs as a function of surround jitter for the mixed, congruent, and incongruent conditions. Each plot is the average slope across observers. Error bars are standard errors calculated across observers.

The differences between the congruent/incongruent and mixed polarity data also find expression in the slopes of the psychometric functions. Figure 6 shows the average across-observer slopes for the congruent, incongruent, and mixed polarity conditions. Whereas the slopes for the congruent and incongruent conditions appear to increase systematically with surround jitter, those for the mixed condition are less consistent but nevertheless show an overall decrease.

To examine whether the psychometric function slopes for the mixed, congruent, and incongruent conditions are different, we carried out a two-way repeated-measures ANOVA with the factors Condition (congruent vs. incongruent vs. mixed) and Surround Irregularity (9 levels). The analysis revealed a significant main effect of stimulus condition (F(1.43, 8.59) = 23.2, *p* < 0.001, $\eta_{p}^{2}$ = 0.8). However, the main effect of irregularity (F(3.16, 18.9) = 2.46, *p* = 0.092 $\eta_{p}^{2}$ = 0.29) and the interaction effect between the condition and irregularity (F(3.96, 23.7) = 2.54, *p* = 0.067, $\eta_{p}^{2}$ = 0.3) were not significant. Bonferroni corrected post-hoc comparison (paired-samples *t*-tests) between stimulus conditions showed significant differences between congruent and mixed (*p* = 0.01, 95%CI [3.49 19.8]), incongruent and mixed (*p* = 0.005, 95%CI [5.56 22.201]) conditions but no significant difference between congruent and incongruent slopes (*p* = 0.443, 95%CI [−6.596 2.165]).

## 4. Discussion

**Figure 7:**
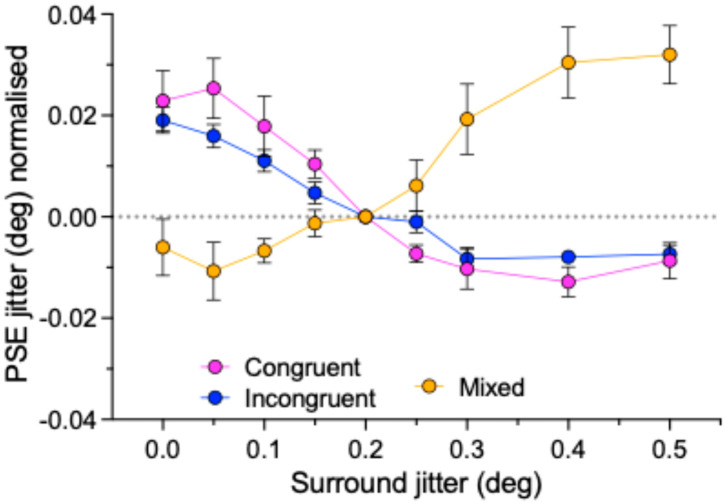
Summary of normalized data averaged across 7 observers. Error bars are standard errors calculated across observers.

Simultaneous regularity contrast (SRC) was demonstrated in Gaussian dot textures made from uniform-in-polarity surrounds and tests, consistent with a previous report (Sun et al., 2019) [10]. On the other hand, an assimilation effect was found with the mixed polarity center and surround conditions. Figure 7 summarizes the main results of the study by showing the relative amounts of normalized data for the congruent, incongruent, and mixed polarity conditions.

In keeping with other texture dimensions, such as density [25,40], SRC is bidirectional. We measured perceived regularity for tests with 0.2 deg jitter with surround regularities ranging from 0–0.5 deg jitter. The only data available for comparison come from Sun et al.’s [10] measurements of the equivalent of our congruent conditions. For comparison, we calculated the size of SRC for the unnormalized congruent conditions as the percentage change from the peak to the trough PSEs. For our data, the value is around 20% which is comparable to Sun et al.’s [10] value of around 18%.

### 4.1. Simultaneous Regularity Contrast and Luminance Polarity

One of the aims of the present study was to determine whether SRC is selective for luminance polarity. Using analyses that took into account all the PSE matches across the full range of surround jitters, we found that across observers there was no significant difference in the magnitude of SRC between the congruent and incongruent conditions. On the other hand, when the congruent and incongruent conditions were fitted separately with a sigmoidal function, resulting in R^2^ values of 0.99 and 0.98, respectively, the two fits were significantly different. We are, therefore, left with a quandary: no difference in the magnitude but a difference in the pattern of SRC between the congruent and incongruent conditions.

The lack of a significant difference in the magnitude of SRC between the congruent and incongruent conditions, like any null result, could, however, be due to a range of factors, such as an insufficient number of practice and/or experimental trials (although note the convergence of PF fitting procedures) or an insufficiently sensitive statistical test of SRC. These mitigating factors are consistent with our finding of the small, albeit non-significant difference between the congruent and incongruent SRC magnitudes, as evidenced by the linear regression fits (top left panel of Figure 5), which show a steeper negative slope for the congruent compared to incongruent data. Although the slope difference, which is based on the average across-observer data, is not significant (*p* = 0.1959), if one repeats the analysis by fitting regression lines to each observer, the difference becomes significant (F(1, 122) = 4.789, *p* = 0.0305). However, the goodness-of-fit R^2^ values for the all-observers congruent and incongruent data, 0.63 and 0.68, are worse than the 0.81 and 0.85 values for the average across-observer data, so we adopt the more conservative position that aligns with the ANOVA results, namely a null result.

Turning now to the significant difference in the pattern of SRC between the congruent and incongruent conditions revealed by the sigmoidal fits (Figure 5), it is possible that the difference in the pattern of SRC between the congruent and incongruent conditions reflects an attentional-based mechanism. Studies examining luminance/polarity selectivity in other types of image regularities, such as symmetry, have shown that although *sensitive* to luminance polarity/color, symmetry mechanisms are not *selective* to these features [41,42] but rather modulated by feature-based attention [41]. It is possible that the small difference in the pattern of SRC between the congruent and incongruent conditions reflects the extra attentional load involved in segregating centre and surround in the former condition.

The significant differences in the sigmoidal fits are nevertheless consistent with the presence of two processing streams, one comprised of ON and OFF cells whose responses are pooled prior to the computation involved in SRC and another in which they are kept separate. If so, the results of the present study are at odds with those of Yamada et al. [9], whose study of the “randomness” after-effect found no significant evidence for polarity selectivity. However, Yamada et al. [9] did not report individual data from the six observers tested, making it difficult to evaluate whether or not there was any consistency in the pattern of responses across observers and/or conditions.

### 4.2. Assimilation with Mixed Polarity Textures

Why does assimilation not contrast occur with our mixed dot polarity textures? A possible tell-tail sign is the relative difficulty one experiences in segregating the test from surround in the mixed compared to the congruent/incongruent conditions (see Figure 1). Although our observers were instructed to maintain fixation on the center of the stimulus, it is possible that neural receptive fields straddled the test/surround boundary, shifting the perceived regularity of the test region toward rather than away from that of the surround. As an anonymous reviewer pointed out, we might expect that assimilation would switch to contrast with either a larger test/surround gap or with a single polarity surround combined with a mixed polarity center (and/or vice versa). We plan to investigate these possibilities in a future study that manipulates the size of the gap in each of the present polarity conditions plus conditions with center/surround combinations of mixed and single polarities. A counterargument, however, to the ‘receptive field straddle’ explanation is that one would expect this to occur also for the congruent (all white or all dark center/surround) conditions where the opposite direction of surround induction to that of the mixed stimuli was found.

An altogether different explanation for the mixed polarity results is that the mixture of polarities disrupts the mechanisms involved in processing regularity, as expected if those mechanisms were reliant upon the presence of polarity-coherent groupings of texture elements. Polarity-coherent groupings of texture elements appears to be an important factor in the aforementioned domain of symmetry perception. Such groupings facilitate symmetry perception when symmetry is not readily salient yet inhibit symmetry perception when luminance polarity and symmetry axes are not aligned [43]. In relation to the present study, a reliance on polarity-coherent groupings would be expected if regularity processing involved spatial-frequency- and/or orientation-selective cortical neurons whose receptive-field sub-regions were themselves selective for the luminance polarities of their LGN afferents. Note, however, that this explanation does not preclude the possibility that at a later stage of processing the lateral interactions between surround and center that underpin SRC could themselves be agnostic to luminance polarity.

### 4.3. Model of Simultaneous Regularity Contrasts

**Figure 8:**
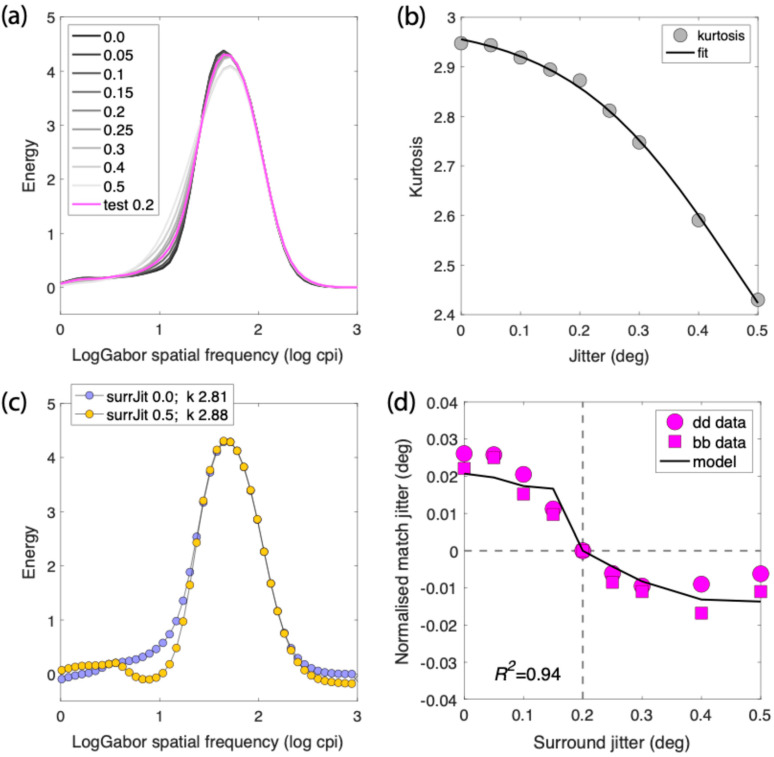
Model of simultaneous regularity contrast. (**a**) RMS distribution of filter responses across log Gabor spatial frequency as a function of the 9 jitter levels of our stimuli, with jitter level increasing from black to mid-grey. The test jitter distribution is shown in magenta. (**b**) kurtoses of the distibutions as a function of jitter, fitted with an exponential function (Equation (3)). (**c**) resulting distributions of the test following non-weighted (for illustration purposes) subtractive inhibition (Equations (1) and (2)) of the 0.0 jitter (blue) and 0.5 jitter (yellow) surrounds, with kurtoses given in the legend. (**d**) data and continuous line model fit using weighted subtractive inhibition.

In this section, we provide a quantitative model of SRC inspired by a suggestion from Sun et al. [10]. Before proceeding to the model of SRC, however, we consider how regularity per se is likely encoded. The observation that different degrees of regularity can be effortlessly discriminated (see Figure 1) has led investigators to suggest that pattern regularity is a texture dimension mediated by Fourier (or wavelet) energy-based operations [8,10,33,34]. As we noted in the Introduction it has been suggested that pattern regularity could therefore be encoded by the “peakedness” in the population response of channels selective for spatial frequency (SF). To illustrate how regularity might be computed from SF-peakedness, we performed an SF analysis similar to that of Ouhnana et al. [8] and Sun et al. [10,34] for the 9 regularity levels in our stimuli. We used square images of 22 × 22 dots for each regularity. The stimuli were filtered by 47 odd-symmetric Log Gabor filters ranging from 0.7 to 1024 cycles per image, each at 4 orientations (0, 45, 90, and 135 deg). The pixelwise root mean square (RMS) responses (i.e., energy) of each filtered image were normalized by the square of the filter size, and the RMSs were then averaged across orientation. The results, plotted in Figure 8a, show that as one proceeds from high to low regularity (dark-to-light lines), the SF distribution becomes less peaked, i.e., more spread out. Kurtosis captures the peakedness of the distribution, and Figure 8b shows that kurtosis declines as jitter increases (i.e., as regularity decreases).

As also noted in the Introduction Sun et al. [10] argued that SRC might be a consequence of within-SF-channel surround-to-center inhibition. In principle, the inhibition could occur for any SF channel whose amplitude is higher in the surround compared to that of the center; that is, the inhibition is “unidirectional”. In the present model, the inhibition is set to be proportional to the weighted difference in amplitude contrast between surround and center.

If *S*(*j_s_,f*) and *T*(*j_t_,f*) are the RMS distributions for, respectively, the surround jitter level $j_{s}$, test jitter level *j_t_* (fixed at 0.2), and spatial frequencies *f*, as shown in Figure 8a, the magnitude of surround inhibition *I* of the test *T* is given by:

(1) $$I\left(j_{s},j_{T,}f\right)=\frac{S\left(j_{s},f\right)-T(j_{t},f)}{S(j_{s},f)+T(j_{t},f)}\;if\;S\left(j_{s},f\right)>T\left(j_{t},f\right)\;else\;I\left(j_{s},j_{t,}f\right)=0$$

Let $T\prime (j_{t},\;f)$ be the RMS distribution of the test stimulus after inhibition, then:

(2) $$T^{\prime }\left(j_{s},f\right)=T\left(j_{t},f\right)-wI\left(j_{s},j_{t,}f\right)$$

where *w* is the weight or gain of the inhibition. Figure 8c shows plots of $T\prime (j_{s},f)$ for surround jitters $j_{s}$ = 0.0 and 0.5, i.e., for the two ends of the surround jitter range, with *w* set to unity for the purpose of illustration (the value of *w* that fitted the data was much smaller—see below). As shown in the figure, the kurtoses *k* of the two post-inhibition test distributions with *w* = 1 are, respectively, 2.81 and 2.88.

The final modeling stage is to determine the match jitters that give the same kurtoses as the 9 post-inhibition tests. To this end, we fitted the following exponential function to the kurtosis measurements in Figure 8b, shown as the continuous line through the data in the figure.

(3) $$k=a+\frac{1}{1+{exp}^{-b(c-j)}}$$

where *k* = kurtosis and *j* = jitter, with fitted parameters *a* = 1.99, *b* = 7.15, and *c* = 0.46. To make the prediction of SRC, we invert Equation (4) to obtain the function that gives jitter as a function of kurtosis:

(4) $$j_{m}=c+\frac{ln\left\lceil \frac{1}{k-a}-1\right\rceil }{b}$$

where $j_{m}$ refers to the jitter of the match stimulus. We hand-fitted the model to the bb and dd data, the simplest conditions without polarity effects. To achieve an acceptable fit, we incorporated a small accelerating nonlinearity with an exponent *e* of 1.008 applied to the RMS distributions in Figure 8a and set the value of *w* to 0.15. After applying the same normalization as applied to the data, the result is the continuous but unsmoothed line through the data shown in Figure 8d. Apart from the three parameters *a*, *b*, and *c* used for generating the continuous line in Figure 8b, the data are fit with just two free parameters, *e* and *w*.

The model fit to the data gives a coefficient of determination R^2^ of 0.94. Although this is a reasonably good fit, the model is inevitably an over-simplification of the computations involved, as there are doubtless other nonlinearities in the transduction of the stimuli, the computation of surround inhibition, and the coding of kurtosis. Moreover, there are statistical measures besides kurtosis that could mediate the perception of regularity, such as the standard deviation of the post-inhibition test SF distributions, as described in Sun et al., 2019 [10]. In these regards, the model proposed here is best considered a “proof of concept”.

## 5. Conclusions

In keeping with one previous report, we have demonstrated that simultaneous regularity contrast (SRC) in dot textures is bidirectional. Luminance polarity differences between surround and test regularities had a small but non-significant effect on the overall magnitude of SRC but a significant effect on the pattern of SRC across surround regularity, suggesting an involvement of both polarity-selective and non-selective mechanisms and/or a contribution of feature-based attention to the center/test polarity. A model involving spatial-frequency-selective surround inhibition with regularity encoded by the peakedness in the distribution of spatial-frequency selective responses gave a good account of the data.

## Data Availability

The original contributions presented in this study are included in the article. Further inquiries can be directed to the corresponding author.

## Appendix A

To confirm the presence of SRC in the congruent and incongruent conditions, we carried out directional one-sample t-tests on the normalized PSEs to examine whether the surround induction was significantly greater/smaller than zero, for each surround jitter condition and irrespective of the centre-surround polarity combination. *d* = cohen’s *d*, a measure of effect size. CI = confidence interval.

Surround jitter

0	t(27) = 8.078, *p* < 0.001, *d* = 1.527, 95% CI [0.016 Inf]
0.05	t(27) = 8.093, *p* < 0.001, *d* = 1.529, 95% CI [0.016 Inf]
0.1	t(27) = 5.016, *p* < 0.001, *d* = 0.948, 95% CI [0.009 Inf]
0.15	t(27) = 3.974, *p* < 0.001, *d* = 0.751, 95% CI [0.004 Inf]
0.25	t(27) = −2.133, *p* = 0.021, *d* = −0.403, 95% CI [−Inf −0.0001]
0.3	t(27) = −4.841, *p* < 0.001, *d* = −0.915, 95% CI [−Inf −0.006]
0.4	t(27) = −6.411, *p* < 0.001, *d* = −1.212, 95% CI [−Inf −0.008]
0.5	t(27) = −5.139, *p* < 0.001, *d* = −0.971, 95% CI [−Inf −0.005]

## Appendix B

To confirm the presence of assimilation in the mixed-polarity condition, we carried out directional one-sample t-tests on the normalized PSEs to examine whether surround induction was significantly greater/smaller than zero, for each surround jitter condition. Abbreviations as in Appendix A.

Surround jitter

0	t(6) = −1.077, *p* = 0.161, *d* = −0.407, 95% CI [−Inf 0.005]
0.05	t(6) = −1.875, *p* = 0.055, *d* = −0.709, 95% CI [−Inf 0.004]
0.1	t(6) = −2.824, *p* = 0.015, *d* = −1.067, 95% CI [−Inf −0.002]
0.15	t(6) = −0.485, *p* = 0.323, *d* = −0.183, 95% CI [−Inf 0.004]
0.25	t(6) = 1.217, *p* = 0.135, *d* = 0.460, 95% CI [−0.004 Inf]
0.3	t(6) = 2.765, *p* = 0.016, *d* = 1.045, 95% CI [0.002 Inf]
0.4	t(6) = 4.354, *p* = 0.002, *d* = 1.646, 95% CI [0.016 Inf]
0.5	t(6) = 5.554, *p* < 0.001, *d* = 2.099, 95% CI [0.021 Inf]
